# Factors Influencing the Implementation of Foreign Innovations in Organization and Management of Health Service Delivery in China: A Systematic Review

**DOI:** 10.3389/frhs.2021.766677

**Published:** 2021-12-20

**Authors:** Wenxing Wang, Jeroen van Wijngaarden, Hujie Wang, Martina Buljac-Samardzic, Shasha Yuan, Joris van de Klundert

**Affiliations:** ^1^Erasmus School of Health Policy and Management, Erasmus University Rotterdam, Rotterdam, Netherlands; ^2^Institute of Medical Information and Library, Chinese Academy of Medical Sciences & Peking Union Medical College, Beijing, China; ^3^Mohammad Bin Salman College of Business and Entrepreneurship, King Abdullah Economic City, Saudi Arabia

**Keywords:** innovation, implementation, health service, China, systematic review

## Abstract

**Background:** China has been encouraged to learn from international innovations in the organization and management of health service delivery to achieve the national health reform objectives. However, the success and effectiveness of implementing innovations is affected by the interactions of innovations with the Chinese context. Our aim is to synthesize evidence on factors influencing the implementation of non-Chinese innovations in organization and management of health service delivery in mainland China.

**Methods:** A systematic review was conducted according to Preferred Reporting Items for Systematic Reviews and Meta-Analyses (PRISMA) guidelines. We searched seven databases for peer-reviewed articles published between 2009 and 2020. Data were analyzed and combined to generate a list of factors influencing the implementation of foreign innovations in China. The factors were classified in the categories context, system, organization, innovation, users, resources, and implementation process.

**Results:** The 110 studies meeting the inclusion criteria revealed 33 factors. Most supported by evidence is the factor integration in organizational policies, followed by the factors motivation & incentives and human resources. Some factors (e.g., governmental policies & regulations) were mentioned in multiple studies with little or no evidence.

**Conclusion:** Evidence on factors influencing the implementation of foreign innovations in organization and management of health service delivery is scarce and of limited quality. Although many factors identified in this review have also been reported in reviews primarily considering Western literature, this review suggests that extrinsic motivation, financial incentives, governmental and organizational policies & regulations are more important while decentralization was found to be less important in China compare to Western countries. In addition, introducing innovations in rural China seems more challenging than in urban China, because of a lack of human resources and the more traditional rural culture.

## Introduction

The Chinese government has committed to establishing a health system that is accessible, equitable, affordable and efficient for all (1). Improving health service delivery forms a major challenge in achieving these objectives (2). Given the complexity and scale of this challenge, a sequence of incremental improvement steps is unlikely to suffice. Substantial innovations in the management and organization of service delivery are called for (3). Moreover, China has been encouraged to learn from “international best practices” in pursuit of service delivery innovation (4).

Innovation of the management and organization of service delivery has been broadly defined as: “*A novel set of behaviors, routines, and ways of working, that are directed at improving health outcomes, administrative efficiency, cost effectiveness, or users experience and that are implemented by planned and coordinated actions*” (5). China is actively adopting a wide variety of innovative international practices in the management and organization of health service delivery that are novel to China. For example, China is introducing “*family doctors to rejuvenate the three-tier network”* of the Chinese health system, integrating and strengthening primary care in analogy with health systems in Western countries (6, 7). However, the success of implementing such innovations, may depend on unpredictable interactions of the innovations in and with the context (5). For instance, differences in organizational cultures between countries may influence the effectiveness of an innovation (8, 9). Innovations which have been developed in Western healthcare systems may be challenging to implement in China, where the working culture is more collective and the power distance in organizations is much higher (10). At the least, the contextual differences typically imply that adaptation is needed.

Previous systematic reviews have identified factors that influence the spread and implementation of innovations in the management and organization of health service delivery, such as the characteristics of innovations and the system antecedents for innovations (5, 11). However, these reviews are primarily based on Western literature and lack evidence on the spread of innovations in management and organization of health service delivery in other context and from Western to non-Western healthcare systems, such as China.

Several studies on the implementation of innovations in the management and organization of health service delivery in the Chinese context have revealed factors influencing the success of implementation, such as knowledge & skills of those involved and their awareness of the innovation and their roles in the implementation (12–14). However, present knowledge and understanding are scarce and fragmentary. To gain a systematic understanding of factors that influence the successful implementation of such innovations from abroad in China, we conducted a systematic literature review. The main research question of the systematic review was: what factors influence the implementation of innovations in management and organization of health service delivery that are developed abroad and implemented in China?

## Methods

### Search Strategy

The following seven databases (4 English and3 Chinese) were systematically searched for eligible studies: Embase, Medline ALL Ovid, Web of Science Core Collection, Cochrane CENTRAL register of trials, CNKI, VIP, and WANFANG. We restricted the year of publication to 2009 and later because in 2009 China initiated a new round of national health-care reform in which it explicitly intended to adopt international best practices (10, 15). The design of the English and Chinese literature searches was supported by a Dutch and a Chinese librarian respectively to promote equivalence of the search strategies. The search strategies are included in Supplementary Material 1.

### Eligibility Criteria

Studies which met all criteria below were included:

- Address innovations related to the organization and management of health service delivery;
- The innovations originate from abroad;
- The innovations are implemented in mainland China;
- The studies present factors influencing the implementation;
- Presents original empirical research;
- Published in a peer-reviewed scientific journal;
- Published between 2009-2020, with all data collected in 2009 or later;
- Written in English or Chinese.

### Record Selection

The screening process consisted of two steps. In the first step, three researchers (WW, JW, and WH) independently screened all English-written articles (WW and JW) and Chinese-written articles (WW and WH) by scanning the titles and abstracts. Articles were excluded if they did not meet all inclusion criteria. If disagreement existed, the articles were then screened by a third reviewer (JvdK: English and YS: Chinese) who had a decisive vote. The origin of the innovations was not always explicitly specified. In such cases, the first author performed an internet search for additional information. Based on this information, articles were excluded if there was consensus among three reviewers (WW, JW, JvdK) that the innovation was not from outside China. In the second step, three reviewers (WW, JW, and WH) independently screened the articles by closely reading the full texts. In case of disagreement, the same third reviewers (JvdK, YS) made the final decision.

### Data Extraction and Analysis

Data were extracted using a form which summarized author(s), year of publication, innovation, study aims, context, innovation origin, study design, factors presented as study results, factors proposed otherwise (e.g., mentioned in the introduction or discussion), and conclusions. As the Chinese context and our cross-border innovation focus are essentially different from the Western contexts of the evidence considered in preceding reviews and on which existing frameworks are based, we adopted an inductive approach. This avoids limitations imposed by deductively following frameworks whose validity is not established for the Chinese context. Moreover, it naturally allows identifying factors and categories for which apparently no evidence has been identified in Western reviews but which are relevant in the Chinese context (5, 11, 16). Following the approach of other systematic reviews to identify factors from a heterogenous set of qualitative and quantitative studies, we conducted a narrative synthesis (17, 18). In our study, two authors (WW and JW) conducted inductive data analysis independently, in the form of close reading of all included studies to identify factors and categories of factors. The identified factors were categorized, synthesized and discussed in various inductive cycles until consensus was reached. Furthermore, the two other authors (MB, JvdK) were involved to assess the consistency and logic of these factors and categories, resulting in three more improvement iterations until final consensus among all authors.

### Quality Assessment of Included Studies

The heterogeneity of the included studies brought along a wide variety in research design and quality (19). We assessed the level of the evidence presented using the classification of the Oxford Center for Evidence Based Medicine (OCEBM level) (20).

## Results

**Figure 1:**
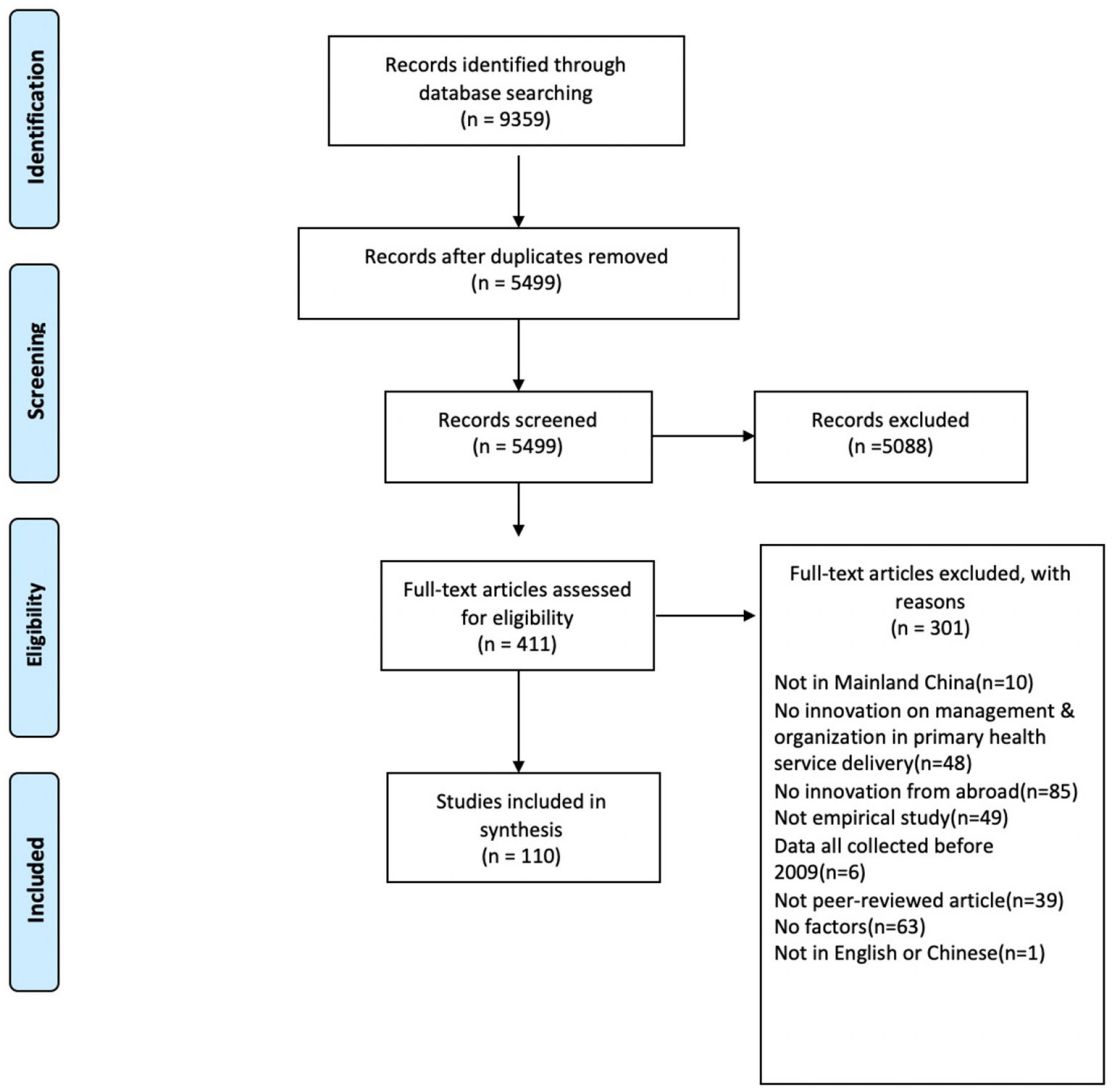
PRISMA 2009 flow diagram.

The search strategy resulted in 5499 articles after 3860 duplicate records were removed. Of these, 417 met all inclusion criteria in the first step and were included for full text screening. After the second round, 110 articles were included for the review. Figure 1 shows the PRISMA flow diagram. Before reporting on the findings, we present a synthesis of descriptives on the included studies.

More than one-third of the studies (*n* = 43, 39%) presented evidence on factors influencing the implementation of the innovation in the study results (see Supplementary Material 2). Almost two-third of the studies (*n* = 67, 61%) discussed possible factors without presenting evidence (see Supplementary Material 3). The 43 studies with evidence mostly had quantitative designs (*n* = 27, 24.5%), while some had a qualitative design (*n* = 11, 10%) or used mixed methods (*n* = 5, 4.5%). Following the OCEBM evidence classification, most studies (*n* = 106, 97%) presented level 3, 4 or 5 evidence. The quantitative studies that used stronger designs (level 1 or 2) were not designed to identifying factors influencing the implementation of the innovation and discussed such factors rather than presenting evidence as a result of the research conducted.

The reader may also find the descriptive synthesis of the wide variety of innovations introduced in the included studies presented in Supplementary Material 4 helpful to develop an impression of the studies and evidence included. It categorizes each of the studies, where each study is categorized only once even it may fit in multiple categories. The main categories are evidence-based best practices (*n* = 42, 38%), integrated care approaches (*n* = 26, 24%) and management tools (e.g., balanced score card) (*n* = 26, 24%).

The majority of the included studies was conducted in Chinese public hospitals (*n* = 71, 65%). From the studies specifying the country from which the innovation originated (*n* = 41, 37%), most came from Australia (*n* = 16,15%) and the United States (*n* = 16, 15%), followed by Japan (*n* = 5, 5%). Most studies were conducted in the southeast of China (economically developed regions), e.g., in Shanghai (*n* = 29, 26%), Guangdong (*n* = 16, 15%), and Zhejiang (*n* = 13, 12%).

Turning from descriptives to the results, let it first be mentioned that the inductive analysis of the 110 included studies resulted in 33 factors and seven categories. Table 1 presents the factors per category, with a description of each factor. Below, we report on the findings of the most frequently mentioned factors per category and provide more details on findings which are specific for China. Table 2 summarizes the main findings with supporting evidence.

**Table 1:** The list of factors and descriptions.

**Category**	**Factor**	**Description**	**Studies presenting evidence-supported factors**	**Studies presenting factors without supporting evidence**
Context	Culture fit	Poor alignment with Chinese cultural norms, values, and thinking acts as a barrier to adoption and implementation of the innovation.	(21–24)	(25, 26)
Context	Doctor-patient relationship	Tensions between doctors and patients, resulting from lack of trust of patients in the competences of the doctors and conversely, lack of trust by doctors in the cooperation of the patient. Moreover, doctors may fear litigation resulting from implementing the innovations in which patients are directly involved.	(12, 14, 22, 27–29)	(25, 30–34)
Context	Resource scarcity in rural areas	Limited availability and accessibility of qualified resources in rural areas, regarding hardware, facilities, and healthcare staff.	(21, 35)	(29, 36–38)
System	Governmental policies and regulations	Policies, systems, regulations and laws available to support the implementation of a certain innovation; e.g., a referral system.	(21, 28, 39, 40)	(14, 23, 36, 37, 41–52)
System	Health insurance	Health insurance coverage and reimbursement rates of the treatments that form the innovation, to promote adoption and sustainability of the innovation. Health insurance affects out-of-pocket expenses for the patient which may influence uptake.	(22, 28, 29)	(51, 53)
System	Hospital level system in China	Higher level hospitals in China's three-tier hospital systems often are more attractive to both patients and health professional. They tend to be more open to and capable of adopting and implementing the innovation.	(17, 54, 55)	(56)
System	Health system stakeholders	As a salient stakeholder, the government plays the central role in China's health system in complex interactions and therefor may have much influence on implementation of innovations. Other stakeholders such as the medical institutions and professionals also have their interests and influence.	(40, 57)	(12, 36, 58, 59)
Organization	Integration in organizational policies	Formal reinforcement by management to integrate an innovation into organizational policies, protocols, etc.	(12, 39, 57, 60–74)	(24, 75–77)
Organization	Workplace culture	Degree to which the workplace culture enables the implementation of innovation.	(78)	(79)
Organization	Strategic fit	Extent to which the implementation of the innovation fits the organizations strategic goals and needs.		(34, 37, 79–81)
Innovation	Clarity	Extent to which the innovation is accompanied by clear instructions and procedures.	(62, 66, 69, 73, 74, 82)	(12, 24, 26, 31, 46, 58, 77)
Innovation	Compatibility	Degree to which the innovation is perceived as consistent with existing work procedures and experience.	(83, 84)	(30, 53)
Innovation	Relative advantage	Extent to which the innovation is perceived as advantageous.	(60, 83)	(25, 85, 86)
Innovation	Risk	Degree of uncertainty of the outcome of the innovation.		(62, 87)
Innovation	Simplicity	Extent to which the innovation is perceived as simple to use (or user-friendly).	(83, 88)	(49, 89–92)
Innovation	Innovation-workload	Time required for implementing the innovation.	(65, 66, 93)	(49)
Innovation	Costs	Financial costs for adopters incurred by adopting, implementing and sustaining the innovation.		(49, 92)
Users	Knowledge and skills	Knowledge and skills required for implementing the innovation (often requirement for health professionals).	(12, 21, 60, 62, 63, 65, 67, 68, 70, 71, 78, 82, 93, 94)	(30, 77, 95–98)
Users	Motivation and incentives	Intrinsic motivation: the passion and enthusiasm of a health professionals to implement the innovation; Extrinsic motivation: Financial stimuli, consequences, and other incentives (e.g., career advancement) to implement and sustain the innovation.	(12–14, 27, 29, 35, 56, 61, 65, 67, 70–72, 83, 90, 93, 99)	(29, 41, 47, 50, 53, 75, 87, 100, 101)
Users	Awareness	The awareness of users of the existence of the innovation and their role in the implementation.	(13, 14, 29, 67, 70, 71, 83, 93)	(47)
Users	Perception and attitudes	Health professionals' perception and attitudes toward implementation of the innovation.	(14, 41, 58, 63, 69, 70)	(62, 77, 100, 102, 103)
Users	Self-efficacy	Confidence to perform the behavior needed to implement the innovation.	(83)	(25)
Users	Social demographics	Age, marital status, education level, clinical tenure and position, hukou (Chinese residence registration), economic status, and occupation.	(13, 29, 40, 41, 57, 61, 64, 84, 90, 104)	(35, 47, 75)
Resources	Human resources	The number of personnel available for implementing the innovation as well as their capabilities, and their (perceived) current workload.	(12, 14, 21–23, 27, 28, 40, 41, 61, 63, 69, 71, 72, 74, 78)	(32, 36, 38, 46, 75, 77, 87, 94, 95, 103, 105–108)
Resources	Financial resources	Funding available for implementing the innovation.	(22, 23, 28)	
Resources	Materials	Facilities, equipment required for implementing the innovation.	(66, 74)	
Implementation process	Training and education	Standardized and qualified education and training available for health providers when implementing the innovation.	(14, 55, 66)	(29–31, 37, 46, 47, 87, 98, 109–113)
Implementation process	Leadership	Leadership support from higher managers, administrators in the organization, and informal leaders (e.g community leader) with respect to the implementation of the innovation	(21, 22, 28, 61, 63, 72, 73)	(14, 46, 49, 52, 84, 96, 114, 115)
Implementation process	Adaptation	Adaptation of the innovation from abroad to the local, Chinese context. Adaptation of the implementation approach to the local Chinese context.	(14, 22, 28, 35, 39, 56, 62, 93)	(13, 21, 30, 36, 40, 41, 48, 79, 89, 102, 105, 109, 116–121)
Implementation process	Communication and collaboration	Communication and collaboration at different levels to implement the innovation (peer-to-peer, across teams/departments, across institutions).	(12, 23, 39, 40, 57, 61, 72)	(24, 37, 46, 48, 62, 96, 109, 122, 123)
Implementation process	Bottom-up vs. Top-down	Top-down refers to hierarchical policy-making, decision-making, execution and management regarding the implementation of the innovation; while bottom-up refers to a process that is initiated at lower hierarchical levels and traverses upwards.	(28, 40, 61, 72, 99, 124)	(41, 42, 79, 110, 120, 122, 125–127)
Implementation process	Support from relevant actors	Active involvement of partners such as the community, or international organizations when implementing the innovation.	(61)	(44, 87)
Implementation process	Feedback	Users are informed of the effects of the innovation.	(40, 66)	(30, 59, 100, 102, 111, 112)

**Table 2:** Summary of main findings with supporting evidence.

**Factors (number of studies with evidence)**	**Categories**	**Summary on evidence**
Integration in organizational policies (18);	Organization	18 studies show that integrating innovations into organizational polices (*n =* 6) and innovation-related tools and procedures (*n =* 12) (e.g., checklists, tools, systems) by managerial reinforcement, facilitates (otherwise inhibits) the implementation of the innovation.
Motivation and incentives (17);	Users	Intrinsic motivation(*n =* 9) was always mentioned as a barrier in adopting and implementing innovations; often described as “lack of enthusiasm” or “lack of passion”. Extrinsic motivation (*n =* 3) always related to financial stimuli in studies presenting evidence on this factor. Users may be de-motivated if there is negative impact on their personal finance (e.g., income, bonus); 5 studies report that effective incentive mechanisms in organizations form facilitators for the uptake of innovations, of which 3 studies relates to financial incentives.
Human resources (16);	Resources	8 studies provide evidence that the time restrictions felt by health practitioners due to existing clinical workload forms a barrier; 8 studies reported that lack of personnel forms a barrier (e.g., staff, experts) in the implementation of the innovation; 6 studies suggest low capability of health professionals to provide qualified health services.
Knowledge and skills (14);	Users	12 studies report that lack of clinical skills of health practitioners (mostly nurses) forms a barrier for implementing the innovation, while 2 studies raise that patients' lack of knowledge is a barrier especially for those innovations associated with self-management.
Social demographics (10);	Users	10 studies provide evidence that social demographics variables affect implementation. For example, more highly educated users are more likely to adopt the innovation, while lower income is associated with lower uptake of the innovation.
Adaptation (8);	Implementation process	8 studies report that adaptation of the implementation approach is important to the implementation of innovations. Implementation approaches (e.g efficient advocacy, communication) that are not tailored to the Chinese context may hinder the implementation. However, the evidence is not concrete on how and what adaptations needed to be made.
Awareness (8);	Users	8 studies show that if users are not aware of the existence of innovations or their role in implementing the innovation, then the uptake is hindered.
Leadership (7);	Implementation process	5 studies show that support from higher-level managers (e.g., administrators and senior health professionals) in health organizations increases the success of the uptake, implementation and sustainability of the innovation. Another 2 studies raise the importance of informal leaders who can reach potential users via informal networks in implementing the innovation.
Communication and collaboration (7);	Implementation process	4 studies raise that poor peer-to-peer and across-group communication has a negative impact, while 3 studies report that the lack of interdisciplinary or inter-institutional (often vertical e.g., tertiary-primary hospitals) collaborations form a barrier.
Doctor-patient relationship (6);	Context	5 studies report that patients' distrust in community doctors impedes the implementation (especially family-doctor related innovations); 1 study reports doctor's lack of trust in patients' ability to cooperate during the implementation of the innovation as a barrier.
Clarity (6);	Innovation	6 studies suggest unclear procedures or instructions as a barrier. The clarity of the innovation refers to clear instructions, and also to the clear standardization and formality of procedures or instructions. (e.g., “formal” process)
Perceptionand attitudes (6);	Users	6 studies show that resistant attitudes of health professionals' toward the innovation negatively affected the uptake; Perceiving the innovation as unnecessary or to increase the workload also impeded the implementation.
Bottom-up vs. top-down (6);	Implementation process	Top-down decision making appeared to increase the likelihood of success in the earlier uptake(*n =* 2), it sometimes formed a barrier to bottom-up initiatives and adaptation(*n =* 1); while bottom-up has been encouraged, top-down executing and managing may be preferred(*n =* 1). Some evidences suggest adopting a hybrid practice(*n =* 2).
Governmental policies and regulations (4);	System	4 studies show that the support of governmental policies and regulations facilitates the uptake and implementation of the innovation.
Training and education (3);	Implementation process	3 studies report that lack of appropriate training schemes (e.g., clinical training) for health practitioners impedes the implementation of innovations. Moreover, inadequate clinical training, insufficient training systems for general practitioners, practices (rather than theories) training, and training for specified skills (e.g., communication skills) are raised as a barrier.

### Context Factors

This category contains the broad variety of social, cultural, economic, and other contextual factors. Twenty-one studies (19%) reported factors in the context category, identifying the factors cultural fit (*n* = 6, 5%), doctor-patient relationship (*n* = 12, 11%), and resource scarcity in rural areas (*n* = 6, 5%).

First, with respect to the factor of cultural fit, two studies (2%) focused on birth-related innovations in which psychological discomfort experienced by pregnant women concerning institution-based child delivery with the help of midwives found it to be barrier to the implementation of birth-related innovations. The psychological discomfort resulted from the mismatch with Chinese values, related to “accepting suffering” (25) and the tradition of “giving birth at home” in some rural areas (21). In addition, two studies mentioned that Chinese people, especially the elderly, rely on their family to decide about to adopting innovative approaches (22, 26).

Second, the distrust of patients in the skills of doctors and the service quality formed a barrier, especially for family- and community-based innovations (12, 14, 25, 27–33). For instance, in a survey to investigate the factors affecting mutual referral behavior, 69.9% of respondents mentioned the low quality of health service provided by doctors in community health service centers (12). Interestingly, the low trust of health professionals' in the ability of patients to cooperate during and after the innovation implementation also formed a barrier.

A third factor worth highlighting is the resource scarcity in China's rural areas. Compared to urban contexts, rural settings have less high-quality health resources such as facilities, equipment, and staff (21, 29, 35–38). One example was the shortage of skilled birth attendants in rural areas of the Guangxi Zhuang Autonomous Region. This shortage prevented the successful integration of traditional birth attendants into the health system in rural regions, when aiming to provide universal access to skilled birth attendance (35).

### System Factors

This category includes factors related to China' s health system. Combinedly, the 31 corresponding studies (28%) reported the factors governmental policies and regulations (*n* = 20, 18%), health insurance (*n* = 5, 5%), hospital level system (*n* = 4, 4%), and health system stakeholders (*n* = 6, 6%).

First, “policy push” (governmental policy initiatives to promote the innovation implementation) was reported as a supportive factor, especially for innovation involving institutions at multiple levels (37). The support can be achieved by instating specific new policies and regulations (23, 37, 41–44) or by building upon the existing ones (28, 39, 45–47).

China has a 3-tier hospital system, in which hospital levels are set based on their ability to provide medical care, medical education, and to conduct medical research. Higher-level hospitals are more likely to adopt innovations than their lower-level counterparts. For example, regarding implementation of the balanced score card; the differences in the adoption rates between all levels were (very) significant (54).

### Organization Factors

This category includes factors at an organizational level, such as organizational characteristics, cultures, and structures. The corresponding 28 studies (26%) mentioned the factors integration in organizational policies (*n* = 22, 20%), workplace culture (*n* = 2, 2%), and strategic fit (*n* = 5, 5%).

First, 22 studies (20%) reported that implementation was inhibited by the absence of formal reinforcement by management to integrate the innovation with organizational policy/protocols (57, 60–64), or with other innovation-related elements (15, 24, 39, 65–73, 75–77). Those elements include tools (e.g., communication tools to guide nursing handover) (65), checklists (e.g., Chinese HF discharge checklist) (65), indicators (e.g., referral indicators) (12), systems (e.g., information management system) (72), and forms (e.g., ETT: endotracheal tube assessment form) (66).

### Innovation Factors

This category includes the characteristics of the innovation itself. 31 studies (28%) reported on the factors clarity (*n* = 13, 12%), compatibility (*n* = 4, 4%), relative advantage (*n* = 5, 5%), risk (*n* = 2, 2%), simplicity (*n* = 7, 6%), innovation-workload (*n* = 4, 4%), costs (*n* = 2, 2%).

In 13 studies (12%), unclear procedures or instructions related to the innovation were a barrier for implementing the innovation. This was not only related to the clarity of the content of these procedures (e.g., clear workflow on handover procedures) but also to the extent in which procedures were formalized and standardized.

### User Factors

This category includes factors related to the users (health providers and patients) that impact implementation of the innovation. 50 studies (46%) reported on the following factors in this category: knowledge and skills (*n* = 20, 18%), motivation & incentives (*n* = 20, 18%), awareness (*n* = 9, 8%), perception and attitudes (*n* = 11, 10%), self-efficacy (*n* = 2, 2%), and social demographics (*n* = 13, 12%).

Insufficient knowledge and skills regarding the innovation of health professionals (12, 60, 62, 63, 65, 67, 68, 70, 71, 78, 82, 93, 95–98) and patients (21, 30, 60, 77, 94) was raised as an inhibitor of implementing the innovation. For example, in one study (60) nurses' lack of knowledge and skills of stoma management was identified by the project team as one of the barriers. A bundle of interventions, including training, significantly improved compliance rates. Lack of patient knowledge and skills was especially reported for implementation of self-management approaches (21, 30, 77, 94).

Second, the factor motivation & incentives was mentioned in multiple studies both in terms of intrinsic (*n* = 15, 14%) and of extrinsic (*n* = 5, 5%) motivation. The former one relates to an individual's passion and enthusiasm to implement the innovation. For example, nurses' lack of enthusiasm to implement discharge education formed a barrier when implementing discharge planning for acute coronary syndrome patients (67). Extrinsic motivation mostly related to financial incentives in the included studies. For instance, frontline doctors were reluctant to adopt clinical pathways when their bonus incomes which are based on drugs and services prescribed were affected (99). Likewise, nurses would choose comprehensive hospitals (59.7%) and tertiary hospitals (51.9%) for dual practice (multiple job-holding) over working solely for primary health care centers (41) because of financial advantages. In addition, six studies (6%) reported that incentive mechanisms in organizations formed facilitators for the uptake of innovations, among which half could be characterized as financial incentives (27, 56, 72). For instance, 88 of 89 hospitals used the balanced score card for improving financial results by linking performance to the assignment of bonuses (56).

Finally, awareness of the innovation and of one's role in implementing the innovation was identified as a factor in nine studies (8%) (13, 14, 29, 47, 67, 70, 71, 83, 93). For example, awareness of the family doctor policy had significant effect on residents' decision to sign family doctor contracting services (29). Conversely, unawareness of caregivers regarding their role in helping patients adhere to fluid-intake restrictions was identified as a barrier to implementation (93).

### Resource Factors

This category includes various resources required to implement innovations. The 31 studies (28%) in this category reported the factors human resources (*n* = 30, 27%), financial resources (*n* = 3,3%), and materials (*n* = 2, 2%). Human resources were raised as a barrier in case of lack of personnel (*n* = 18, 16%), insufficient capability of health workers (*n* = 10, 9%), or heavy existing workload (*n* = 8, 7%).

First, the lack of personnel (e.g., health workers, experts, coordinators) was raised as a barrier in 18 studies (12, 14, 23, 27, 32, 36, 40, 46, 61, 72, 74, 87, 94, 95, 105–108). Second, existing workload was raised as a barrier for implementing innovations in 8 studies (7%) when the existing workload was perceived to leave no time for implementation of innovations (21, 41, 61, 63, 69, 71, 74, 78). A related barrier is when health professionals perceive that the innovation would result in an increase of workload (14, 41, 58, 62, 63, 69, 70, 77, 100, 102, 103). Finally, the lack of organizational capability; represented by the number of skilled health professionals (often skilled nurses) within health facilities and abilities of qualified health service provision (e.g., emergency departments' capacity to implement thrombolysis) formed a barrier for implementing the innovation in ten studies (9%) (12, 22, 23, 27, 28, 38, 61, 75, 77, 128).

### Implementation Process Factors

This category includes factors that influence the process of turning implementation plans into actions and to succeed with the intended innovation. Factors in this category were reported in 70 studies (64%), including training & education (*n* = 16,15%), leadership supports (*n* = 20,18%), adaptation (*n* = 27, 25%), communication & collaboration (*n* = 16, 15%), bottom-up vs. top-down (*n* = 15, 14%), support from relevant actors (*n* = 3, 3%), and feedback (*n* = 8, 7%).

First, many of these studies identified adaptation as an important factor to facilitate implementation; both the adaptation of the innovation (*n* = 10, 9%) (41, 48, 79, 89, 102, 105, 109, 116–118) and that of the implementation approach. (*n* = 17, 15.5%) (13, 14, 21, 22, 28, 30, 35, 36, 39, 40, 56, 62, 79, 93, 119–121). Four studies (4%) (102, 105, 109, 117), for example focussed on Chinese adaptations of integrated care approaches (e.g., clinical pathways). Clinicians were often responsible for adapting innovations, as was the case when a working group comprising 22 cardiologists representing both level 2 and level 3 hospitals provided expert advice on how to tailor interventions to local circumstances (105). Many studies (16%) reported on the need to adapt the implementation process without further elaboration.

Second, 7 studies reported top-down policy-making and decision-making in China (*n* = 7, 6%) (40–42, 61, 99, 120, 125). While top-down decision making appeared to increase the likelihood of success in the earlier uptake, it sometimes formed a barrier to bottom-up initiatives and adaptation (40, 99, 120, 125). For example, in one study top-down policy goals were not seen as relevant and attainable due to the specific local context (40). Five studies (5%) mentioned bottom-up execution and management to be facilitating (79, 110, 122, 126, 127). While one study (72) reported a top-down approach as a facilitator. Finally, two studies (2%) (26, 124) pointed out that the best practice would utilize both top-down and bottom-up approaches.

Third, leadership was mentioned in 15 studies (14%) as an important facilitator, including high-level administrator support in hospitals (14, 22, 28, 46, 49, 61, 63, 72, 84, 111, 114, 115) and informal leader support (e.g., by community leaders) (21, 73, 96). For instance, many participants perceived making changes to hospital systems or processes based on data audit and feedback as too difficult in the absence of high-level administrative support (22).

Fourth, lack of training & education, especially lack of practical training for health professionals was identified as an inhibitor in 16 studies (15%) (14, 29–31, 37, 46, 47, 66, 87, 98, 104, 109–113). The studies which regarded training programs included both clinical and non-clinical training, such as communication skills training for health care practitioners (30).

Finally, poor peer-to-peer and across-group communication was a barrier (12, 24, 40, 61, 72, 96, 109) while multidisciplinary cooperation and inter-institutional collaborations were of great importance in implementing innovations (e.g., the referral system) (23, 37, 39, 46, 48, 57, 62, 122, 123). For example, close collaboration with other institutions benefited quality and safety improvement projects (46).

## Discussion

This review identified 33 factors in 7 categories that may influence implementation of foreign innovations in management and organization of health service delivery in China. Most supported by evidence were the factors human resources and users' knowledge & skills, hindering implementation because of shortages. Of the 33 factors identified, 28 also appear in the systematic review of Greenhalgh, which is mostly based on western literature (5). Compared to previous reviews which mostly rely on Western evidence (5, 11), new factors or new perspectives relate to motivation & incentives, governmental policies and regulations, integration into organizational policies, clarity of innovations and bottom-up & top-down management approaches.

It is worth noting that out of the 110 studies included only 43 present results with evidence on factors. Moreover, 29 of these 43 studies were not specifically designed to identify factors, but for example focused on the outcomes achieved. Nevertheless, many factors were mentioned in multiple studies, suggesting they are relevant. For example, governmental policies & regulations to support implementation were mentioned in 20 studies, four of which provided evidence. Likewise, the need to adapt the innovation and the implementation methods to the (Chinese) context was mentioned in many articles, mostly without supporting evidence or specifics.

Western evidence suggests that both intrinsic and extrinsic motivation can stimulate potential users of an innovation (5). It is important for the innovation to meet an identified need of the user (129) and costs and benefits of the innovation should be balanced (5). For instance, several theoretical frameworks indicate that user's may be willing to put in extra time and effort by utilizing an innovation, if it improves the quality of their work (130). However, our review only provided evidence for intrinsic motivation as a barrier (e.g., lack of enthusiasm) for implementation. Extrinsic motivation, especially based on financial incentives, was identified as both a barrier and facilitator; potential financial gains or losses were likely to motivate or de-motivate users. This confirms other Chinese studies that show how intrinsic rewards, compared to extrinsic and social rewards, have limited influence in public sectors in China compared to the West (131). Research also found that career development and financial benefits rank first and second in motivating health workers in China (132). This may be explained by the relatively low incomes and high work-pressure experienced by Chinese health workers (132, 133).

Western evidence furthermore shows that making adoption mandatory may promote the initial adoption of an innovation, yet may decrease the effectiveness and sustainability (5). In contrast, our review provides evidence that formalization (relating s to integration into organizational policies & clarity of innovations in our review) promotes not only the initial adoption, but also the effectiveness and sustainability of innovations. Likewise, both Greenhalgh and Chaudoir (5, 11) state that centralization (relating to top-down and governmental policies and regulations in this review) has a negative effect on innovativeness of organizations, while decentralization to front-line personal (relating to bottom-up approaches) positively affects the implementation of innovations. The evidence we found on decentralization is inconclusive. Some studies suggest that a combination or so called “hybrid” top-down/bottom-up' approach in which leaders initiate top-down procedures that enable and mandate bottom-up initiatives for adaptations are more effective in the Chinese context (10). These differences probably relate to power distance (*the extent to which less powerful members of organizations and institutions accept and expect that power is distributed unequally)* (134). With a power distance index of 80, China scores higher than most Western countries. Chinese *are more likely to be influenced by formal authority and sanctions and are in general optimistic about people's capacity for leadership and initiative)* (134). This may explain why formalization and organizational leadership (relating to top-down leadership) have been attached great importance in the uptake and implementation of innovations in China. Thus, although decentralization has increasingly been encouraged, both governmental guidance and hierarchical superior-subordinate relationship are the norm in China (40–42, 61, 99, 120, 125). The centralized leadership, power and responsibilities defined by authoritative regulations and rules appear to provide users with a sense of certainty and safety, thus facilitating the adoption, implementation and sustainability of innovations.

In addition to cultural differences, the review also revealed other novel, perhaps relatively Chinese contextual characteristics that influence the implementation of innovations. First, availability of skilled personal appears as a major challenge for innovation in China's healthcare system (135). Professionals either lack relevant skills, there is no appropriate training available, or there is a lack of personnel and therefore time. Most included studies are about innovations in tertiary hospitals which have more highly skilled staff. If human resource capacity is mentioned as a barrier in tertiary hospitals, it likely applies even more to primary care, for which the evidence however remains very scarce.

A second specifically Chinese contextual finding relates to the challenges encountered in rural areas. The few studies on innovation in rural China mention challenges faced in relation to the resource disparity compared to urban areas. With China's urbanization, about half a billion people moved from rural areas to urban areas over the past three decades (136). Corresponding reallocations of labor across space and sectors in some parts of China contributed to rapid economic growth but increased disparities between rural and urban areas (136). As many skilled health professionals have been attracted to big cities, availability of qualified health services in rural areas tends to be restricted (135). In addition, cultural norms in rural China are more traditional, which may form a general barrier for innovation (10). Such rural challenges underlie several of our findings, e.g., regarding innovations in maternal and child health services (21, 25).

## Limitations

As there is no universally adopted terminology or strong indexation in both Chinese and English databases for the concept of innovations in organization and management of health service delivery, it is possible that some published studies meeting the inclusion criteria were not identified through the search. In addition, although we designed the search in a librarian-mediated and a process-equivalent manner, some linguistic differences between Chinese and English may not have been addressed. We found that concepts (notably innovation itself) have no direct translation from English to Chinese. We therefore used an adapted combination of search terms in Chinese databases. Also, Chinese databases use different algorithms compared to English databases to facilitate searches, for example they often do not use mesh terms. The assistance of librarians helped us to navigate both Chinese and English database to try and find similar types of publications, but differences may have remained.

Our synthesis of the evidence is limited by the lack of studies with robust evidence and the large heterogeneity in methods used, innovations studied, and aims. Few studies explicitly focused on identifying factors that influence implementation of innovations in China. Furthermore, most studies were done in more affluent parts of China and primarily in tertiary hospitals with better skilled staff. As our analysis has not compared innovations from abroad with domestic innovations, caution is called for when attributing the results to the non-domestic origin of the innovations considered.

## Implication for Research and Practice

There is a general lack of studies on implementation processes of innovations in the Chinese health system. Most studies are quantitative in nature and report primarily on the relationship between an innovation and clinical outcomes, while the implementation process remains a black box. A better understanding of these processes not only requires studies with more robust designs, but also qualitative studies to identify why and how factors frequently mentioned in included studies affect the uptake and implementation of innovations. Furthermore, these studies should also include rural areas in China and primary care facilities.

By studying which factors influence the implementation of innovations from abroad in China we tried to better understand contextual influences. Many of the identified factors also surfaced in previously conducted reviews which are strongly biased toward affluent Western countries with individualistic working cultures and relatively low power distance. This review provides first evidence that such contextual differences between countries may have more influence on implementation of innovation processes than has been recognized. Previous reviews have largely disregarded such cultural and country-related differences.

Our review presents new factors and provides new perspectives on previously identified factors. This evidence base can serve to advance further research and better inform policy and practice to implement international best practices to achieve China's health reform objectives. First, it is of practical importance to bear previously identified factors in mind while implementing foreign innovations in organization and management of health service delivery, and additionally consider the factors especially important in China such as extrinsic motivation, financial incentives, governmental and organizational policies & regulations. Moreover, it is important to recognize that, somewhat in contrast with existing evidence from Western contexts, centralization and top down mechanisms promote implementation in the Chinese context with high power distance. Lastly, it is important to recognize that China is not a small monocultural context. Within the large country of China, considerable differences exist, for instance between rural and urban China. The local characteristics must be addressed when implementing innovations.

## Conclusion

This review shows that China has specific cultural and contextual characteristics that need to be taken into account when implementing innovations in management and organization of health service delivery from abroad. Compared to many of the countries in which these innovations originate, China has a high-power distance. Formalization and organizational leadership are therefore more important for successful implementation. Furthermore, financial incentives are prime motivators in China. Also, China has major shortages in skilled health personal, on top of large disparities in development and capacity between primary and secondary care and between affluent and less affluent regions. Implementation of innovations is therefore much more challenging in primary care and developing areas in China. Although many factors identified in this review are not specific for innovations in China, our findings suggest that cultural and contextual differences have more impact than is currently recognized in literature.

## Data Availability Statement

The original contributions presented in the study are included in the article/Supplementary Material, further inquiries can be directed to the corresponding author/s.

## Author Contributions

SY provided support in finalizing the Chinese search query. WW and JW screened the English titles and abstracts. WW and HW screened the Chinese titles and abstracts. WW, JW, and JK reviewed the English full texts. WW, HW, and SY reviewed the Chinese full texts. WW, JW, MB-S, and JK analyzed the data and categorized the results together. WW initiated the draft of the manuscript. All authors read and approved the final manuscript.

## Funding

This work was supported by China Scholarship Council (CSC201908500106). This funder has no role in the study design, data collection and analysis, interpretation of data and writing the manuscript.

## Conflict of Interest

The authors declare that the research was conducted in the absence of any commercial or financial relationships that could be construed as a potential conflict of interest.

## Publisher's Note

All claims expressed in this article are solely those of the authors and do not necessarily represent those of their affiliated organizations, or those of the publisher, the editors and the reviewers. Any product that may be evaluated in this article, or claim that may be made by its manufacturer, is not guaranteed or endorsed by the publisher.
