# Supplementary material for: Factors Influencing the Implementation of Foreign Innovations in Organization and Management of Health Service Delivery in China: A Systematic Review
Source: Front Health Serv. 2021 Dec 20;1:766677. doi: 10.3389/frhs.2021.766677 (PMC10012679; doi:10.3389/frhs.2021.766677)
Supplement: Supplementary file 1 [file Table_1.docx]

Online supplementary material 2: Studies presented evidence on factors influencing the implementation of the innovation in the study results

| **Author** | **Year** | **Innovation** | **Aim of study** | **Context** | **Study design** | **Factors with supporting evidence** | **Factors without supporting evidence** |
| --- | --- | --- | --- | --- | --- | --- | --- |
| Bai et al.(37) | 2019 | Dual practice (DP) or multiple job‐holding | To identify the intention of nurses to DP and inform policymaking in centralised government settings | Public hospitals  Guiyang, Guizhou | A descriptive multicenter cross‐sectional survey | Human resources Perception and attitudes  Social demographics | Governmental policies and regulations  Motivation  Adaptation Bottom-up vs. Top-down |
| Budd et al. (48) | 2018 | Evidence-based chronic disease prevention (EBCDP) | To better understand the contextual similarities and differences influencing the dissemination and implementation of EBCDP | Community health centers, hospitals, and the Centers for Disease Control and Prevention | Qualitative, semi-structured interviews | Integration in organizational policies Incentives Human resources Social demographics Leadership Communication&collaboration Bottom-up vs. Top-down Support from relevant actors |  |
| Cai and McArthur(77) | 2020 | The Joanna Briggs Institute Practical Application of Clinical Evidence System (JBI-PACES) and Getting Research into Practice (JBI-GRIP)  Evidence-based best practices for discharge planning | To promote evidence-based practice in an endoscopy tertiary center in China and ensure the safe, timely and appropriate discharge of sedated patients from this center | Endoscopy center in tertiary hospital, Shanghai | A before-and-after study based on clinical audit | Integration in organizational policies  Clarity Human resources Materials |  |
| Cao et al.(47) | 2014 | Evidence-based best practices for the management of stoma among colorectal cancer patients  JBI-PACES and GRIP framework | To implement the best available evidence of stoma management into practice and to improve the self-care of patients with a stoma | Colorectal surgical ward of Fudan University Shanghai Cancer Center | A before-and-after study based on clinical audit | Integration in organizational  policies  Relative advantage Knowledge and skills |  |
| Du et al.(12) | 2012 | ‘Mutual referral’ between urban community health serv- ices and hospitals | To evaluate the current status of mutual referral pilot programme, perceived factors that affect referral behaviour and changes that would improve the current referral process in Beijing | Urban district community health service centres in Beijing | A cross-sectional survey | Doctor-patient relationship  Integration in organizational policies Human resources Incentives Knowledge and skills Human resources Communication & collaboration | Health system stakeholders Clarity |
| Furtado et al.(82) | 2018 | EBNCDP | To explore the political barriers to and facilitators of EBNCDP in Australia, Brazil, China and the United States. | Community health centers, hospitals, and the Centers for Disease Control and Prevention in China; | Qualitative semi-structured interviews | Health system stakeholders Social demographics Human resources Governmental policies and regulations  Communication & collaboration Bottom-up vs. Top-down Feedback | Adaptation |
| Gao and Gurd (75) | 2014 | The balanced scorecard (BSC) | To investigate how widely the BSC was being diffused and implemented in Chinese hospitals. | Shangdong Province | A Mixed-methods study (questionnaire survey,  and semi-structured telephone interviews) | Incentives Adaptation | Hospital level system in China |
| Chen et al.(53) | 2016 | The JBI-PACES and GRiP framework   Heart Failure Discharge Checklist | To promote evidence-based discharge planning for heart failure patients in the coronary care unit of Zhongshan Hospital | Coronary care unit of Zhongshan Hospital, Shanghai | A before-and-after study based on clinical audit | Integration in organizational policies  Innovation-workload Knowledge and skills Motivation |  |
| He and Yang (74) | 2015 | Clinical pathways | To present a Chinese public hospital case study where a clinical pathway pilot was undertaken where we evaluate two main outcomes: stay and hospitalization costs for a tertiary hospital from 2010 to 2012 | Public hospital, Shanxi Province | A mixed-methods study: document analysis, interviews | Motivation  Bottom-up vs. Top-down |  |
| Hu et al.(54) | 2017 | Evidence-based best practices in the neonatal intensive care units(NICU)  PACES and GRiP | To implement evidence-based best practice and assess the effects of these strategies on minimizing unplanned extubations NICU | NICU at the Children’s Hospital of Fudan University in Shanghai. | A before-and-after study based on evidence-based best practice | Integration in organizational policies  Clarity Materials Training & Education Feedback |  |
| Huang et al.(13) | 2018 | The “gate-keeper” role of FD (also called the family physician or general practitioner) | To further explore factors associated with contract behavior | Shanghai | A longitudinal survey | Motivation  Awareness Social demographics | Adaptation |
| Jia et al.(66) | 2016 | Evidence-based best practices for the management of non-adherence to fluid intake restrictions in hemodialysis (HD) patients   JBI-PACES and GRIP | To promote evidence-based practice in the management of fluid-intake restrictions among HD patients in a HD center and the status of HD patients on the basis of the best available evidence | Hemodialysis Center of Huashan Hospital, Shanghai | A before-and-after study based on clinical audit | Innovation-workload Knowledge and skills Motivation  Awareness Adaptation |  |
| Jiang et al.(32) | 2016 | Integrated traditional birth attendants (TBAs) and skilled birth attendence (SBA) | To describe and discuss the process of transforming the role of TBAs in China’s Guangxi Zhuang Autonomous Region as a case study, to illustrate how government successfully integrated TBAs into the health system in rural regions | Guangxi Zhuang Autonomous Region (a multi-ethnic mountainous area) | Qualitative in-depth interviews and literature and document reviews | Resource scarcity in rural areas Motivation  Adaptation | Social demographics |
| Lei et al.(84) | 2013 | Electronic medical record systems standards  Meaningful use objectives  US electronic health records (EHR) | To provide valuable information about important EHR functionality to informaticians in the US and China | Peking University First Hospital and Beijing Cancer Hospital | A descriptive study using qualitative data and observation | Perception and attitudes | Health system stakeholders Clarity |
| Li et al.(122) | 2012 | PDCA (plan-do-check-act) cycle | This paper presents the design and development of the EMR, entitled Hygeia, which is specially designed to support medical process management with the PDCA cycle | Healthcare Informatics Engineering Research Center in Zhejiang University | A descriptive study | Simplicity |  |
| Lin et al.(46) | 2014 | The balanced scorecard | This study investigates the outcomes of BSC application in public hospitals contextual to the existing health care administration systems in China | Public hospitals in China | A quantitative survey | Hospital level system in China |  |
| Liu et al.(67) | 2016 | Evidence-based practices for nursing management of post-stroke dysphagia  PACES and GRiP | To improve nursing management of dysphagia in stroke patients and prevent the occurrence of aspiration in patients admitted to the neurology ward of Huashan Hospital, Fudan University | Neurology ward of Huashan Hospital, Fudan University, Shanghai | A before-and-after study based on clinical audit | Clarity Knowledge and skills |  |
| Lu et al.(55) | 2015 | Discharge planning for acute coronary syndrome patients  PACES and GRiP | To improve local practice in discharge planning for acute coronary syndrome patients in Huadong Hospital, Shanghai | Cardiovascular Ward and Coronary Care Unit of Huadong Hospital, Shanghai | A before-and-after study based on clinical audit | Integration in organizational policies  Innovation-workload Knowledge and skills Motivation  Awareness |  |
| Ma et al.(40) | 2015 | HIV/AIDS case management | To explore the challenges and enablers in shifting the HIV/AIDS case management services from Centers for Disease Control and Prevention (CDCs) to Community Health Service Centers (CHSCs) in urban China | CHSCs in three cities (Guangzhou, Nanjing and Changsha) | Qualitative focus groups discussion, in-depth interviews and document analysis | Culture fit Human resources Financial resources Communication & Collaboration | Policies and regulations |
| Ma et al.(22) | 2018 | PDSA( plan-do-study-act) cycles | To test a model which involved clans and health providers to increase antenatal care attendance in rural minority areas of China with high HIV prevalence | Four villages in Zhaojue County, Liangshan Liangshan Yi Autonomous Prefecture | A mixed-methods study (with surveys and in-depth interviews) | Culture fit Resource scarcity in rural areas Human resources Knowledge and skills Governmental policies and regulations | Adaptation |
| Fang et al.(123) | 2018 | Innovative home-based and remote sensing rehabilitation protocol among cardiovascular patients | To present results of investigation on patients’ attitudes towards acceptance of home-based cardiac tele-rehabilitation technology for coronary heart disease | The First Affiliated Hospital of Shantou University Medical College, Guangdong Province | A cross-sectional study | Motivation  Social demographics |  |
| Penm et al.(14) | 2014 | Clinical pharmacy services without additional funds . | To explore the views of hospital administrators, pharmacy directors, clinical pharmacists, and dispensing pharmacists about the factors affecting clinical pharmacy services in China | Public hospitals in four cities (Beijing, Zhengzhou, Luoyang, and Shanghai) | Qualitative interviews | Doctor-patient relationship  Hospital level system in China Motivation  Awareness Perception and attitudes  Human resources Training & education Adaptation | Governmental policies and regulations |
| Qian et al.(63) | 2017 | An integrated care model, namely the Joint Health Center for chronic care | To identify which design features and supporting environments would facilitate or constrain the implementation of integrated care reform in China’s context | Hangzhou, Zhejiang Province | Qualitative in-depth interviews and focus group discussions | Integration in organizational policies  Incentives Human resources Leadership  Communication &collaboration Bottom-up vs. Top-down |  |
| Ranasinghe et al.(23) | 2014 | Clinical pathways in acute coronary syndromes 2 | To understand the problems encountered by health professionals and hospitals in implementing these pathways and specifically to examine the system-level barriers to implementing clinical pathways in the dynamic healthcare environment of China | 75 hospitals throughout China | Qualitative in-depth interviews | Culture fit Doctor-patient relationship  Health insurance  Human resources Financial resources Leadership  Adaptation |  |
| Rosenthal et al.(51) | 2013 | International Nosocomial Infection Control Consortium (INICC) multidimensional hand hygiene approach | To assess the feasibility and effectiveness of the INICC multidimensional hand hygiene approach in 19 limited-resource countries and to analyze predictors of poor hand hygiene compliance | 19 limited-resource countries | An observational, prospective study | Integration in organizational policies  Social demographics |  |
| Su et al.(52) | 2015 | International Nosocomial Infection Control Consortium (INICC) multidimensional hand hygiene approach | To evaluate the impact of the INICC multidimensional hand hygiene approach in three hospitals in three cities of China, and analyze predictors of poor hand hygiene compliance. | Three hospitals in three cities of China | A prospective, before-after multicentric study | Health system stakeholders Integration in organizational policies  Social demographics Communication & collaboration |  |
| Tang et al.(104) | 2017 | Integrated health organization reform | To analyse integrated care reforms using complex adaptive system theory | Qianjiang, a rural district in southwestern China | A mixed-methods study (questionnaires and interviews) | Bottom-up vs. Top-down |  |
| Wang et al.(50) | 2018 | Evidence-based management of cancer related fatigue (CRF)  JBI-PACES and GRiP | To promote evidence-based practice in the manage-ment of CRF for patients in a hospital setting | Oncology unit of Nanfang Hospital, affiliated with the Southern Medical University, Guangdong Province | A before-and-after study based on clinical audit | Integration in organizational policies  Human resources Knowledge and skills Perception and attitudes  Leadership |  |
| Wang et al.(73) | 2019 | The electronic medication monitor (EMM) | To evaluate the acceptance of the EMM among health care workers and patients while implementing the device in actual routine practice by the local TB program. | Zhenjiang, Jiangsu Province | A cohort study using quantitative data | Knowledge and skills | Human resources |
| Wu et al.(68) | 2020 | Evidence-based best practice of managing breast cancer patients  PACES and GRiP | To ensure the provision of comprehensive and structured pre-treatment assessment that supports safe care of patients with breast cancer undergoing chemotherapy | The Breast Surgery Department of tertiary hospital in Guangdong Province | A before-and-after study based on clinical audit | Workplace culture  Human resources Knowledge and skills |  |
| Zhang (56) | 2014 | Evidence-based best practice of management of chemotherapy-induced peripheral neuropathy  PACES and GRiP | To integrate the available evidence of assessment and management of chemotherapy-induced peripheral neuropathy into practice, and implement strategies to improve compliance with acceptable practice. | Chemotherapy Ward, Fudan University Shanghai Cancer Center | A before-and-after study based on clinical audit | Integration in organizational policies  Knowledge and skills |  |
| Xu and Mills (25) | 2017 | Gatekeeping | To make explicit cause-and-effect relationships and facilitate understanding and interpretation of interacting factors and feedback loops that contribute to important policy issues. | Two rural townships in a large municipality in northern China | Qualitative in-depth interviews | Doctor-patient relationship  Incentives Human resources |  |
| Xu et al.(108) | 2019 | Innovative medical consultation model: gatekeeping system  Business rocess reengineering (BPR) | To introduce a new concept of primary care consultation system at a mainland Chinese hospital in response to healthcare reform; and to explore the factors associated with change resistance and acceptance from both patients’ and medical staff’s perspectives | The University of Hong Kong-Shenzhen Hospital, Shenzhen | Quantitative surveys | Compatibility Social demographics | Leadership |
| Yang and Zhang (64) | 2016 | Standardised postoperative handover protocol (included a postoperative handover checklist) | To improve the postoperative handover process and immediate postoperative patient outcomes | The Neurosurgical Intensive Care Unit of a tertiary hospital, Wuhan, Hubei Province | A pretest/post-test study | Integration in organizational policies Clarity Leadership |  |
| Yu et al.(57) | 2017 | Evidence-based best practices in clinical handover in a pediatric setting  PACES and GRiP | To make a contribution to promote evidence-based practices in clinical handovers in a pediatric setting and thereby enhance patient safety and service delivery. | The Gastroenterology Department, Children’s Hospital of Fudan University, Shanghai | A before-and-after study based on clinical audit | Integration in organizational policies  Clarity Human resources Perception and attitudes |  |
| Yuan et al.(26) | 2019 | Family doctor contracting services | To identify the facilitators and barriers to implement family doctor contracting services in China and to shed new light on establishing family doctor systems in developing countries. | 19 primary health institutions in nine provinces purposively selected from the eastern, middle and western areas of China | Qualitative semi-structured interviews | Doctor-patient relationship  Health insurance  Human resources Financial resources Leadership Adaptation Bottom-up vs. Top-down | Policies and regulations |
| Zhang et al.(35) | 2019 | Family doctor signing service | To explore the factors influencing residents' decision to sign with family doctors | Five communities in Xianning, Hubei Province | Quantitative questionnaires surveys | Doctor-patient relationship  Health insurance  Motivation  Awareness Social demographics | Resource scarcity in rural areas Incentives Training & education |
| Zhang et al.(58) | 2017 | Evidence-based practices for postpartum women in managing perineal care  PACES and GRiP | To make a contribution toward promoting evidence-based best practice for postpartum women in managing perineal care, thereby reducing the rates of perineal pain | The Obstetric and Gynecological Hospital, Fudan University, Shanghai | A before-and-after study based on clinical audit | Culture fit Integration in organizational policies  Knowledge and skills Motivation  Awareness Perception and attitudes |  |
| Zhu et al.(59) | 2016 | Evidence-based practices of nursing care for transradial angiography and intervention PACES and GRiP | To assess the implementation rates of evidence-based recommendations regarding nursing care for transradial angiography and intervention in Zhongshan Hospital, Shanghai | Coronary care unit of Zhongshan Hospital, Shanghai | A before-and-after study based on clinical audit | Integration in organizational policies Human resources Knowledge and skills Motivation  Awareness |  |
| Wu (49) | 2014 | Evidence-based best practices of nursing care for laryngectomy patients  PACES and GRiP | To improve the local practice of nursing care for total laryngectomy patients in Shanghai Eye & ENT hospital, and thereby make a contribution towards improving patient outcomes. | Shanghai Eye & Ear Nose and Throat (ENT) hospital of Fudan University in Shanghai | A before-and-after study based on clinical audit | Integration in organizational policies Clarity Knowledge and skills Adaptation | Risk Perception and attitudes  Communication & collaboration |
| Su et al.(124) | 2018 | Evidence-based pain management practices | To investigate the practice level of evidence-based pain management in head nurses, and explore influencing factors of implementing pain management | 257 hospitals of Hunan Province | Quantitative surveys | Hospital level system in China Social demographics Training & education |  |
| Yuan et al.(45) | 2015 | Innovative care for chronic conditions | To explore key factors influencing the the chronic disease management in community health service institutions | 12 community health service centers in four cities (Beijing, Shanghai, Zhengzhou and Chengdu) | Qualitative interviews | Governmental policies and regulations Integration in organizational policies  Adaptation Communication & collaboration |  |
| Zhao (76) | 2017 | Mobile Health Management Service (MHMS) | To investigate factors influencing the use intention of MHMS | Research Center on Information Resource of Wuhan University, Hubei Province | A Quantitative survey | Compatibility Relative advantage Simplicity Motivation  Awareness Self-efficacy |  |
