# Supplementary material for: Factors Influencing the Implementation of Foreign Innovations in Organization and Management of Health Service Delivery in China: A Systematic Review
Source: Front Health Serv. 2021 Dec 20;1:766677. doi: 10.3389/frhs.2021.766677 (PMC10012679; doi:10.3389/frhs.2021.766677)
Supplement: Supplementary file 2 [file Table_2.docx]

Online supplementary material 3: Studies discussed possible factors without presenting evidence

| **Author** | **Year** | **Innovation** | **Aim of study** | **Context** | **Study design** | **Factors without supporting evidence** |
| --- | --- | --- | --- | --- | --- | --- |
| Bai et al.(85) | 2018 | Clinical pathways(CPs) | To determine the effects of CPs on medical care in the hospital setting, including the perceived effects of CPs on medical care and objectively measured patient outcomes | 54 public hospitals in Shanghai, Hubei, Gansu Provinces | A quantitative survey and chart review | Perception and attitudes  Adaptation Feedback |
| Browning et al.(28) | 2015 | Chronic disease self-management program (the Happy Life Club™): 12-month diabetes management program | To report data based on a 12-month pilot study  To discuss issues involved in the implementation of Chronic Diseases Self-Management Programs in China | Fangzhuang community, Beijing | A cluster randomized controlled trial | Doctor-patient relationship  Compatibility Knowledge and skills Training & education Adaptation Feedback |
| Cao et al.(60) | 2019 | The Evidence-based Practice for Improving Quality (EPIQ) | With the objective of improving neonatal outcomes, we have adopted the EPIQ method and started systemic practice changes in a single center in China since 2008 | Neonatal intensive care units, Fudan University, Shanghai | A before-and-after study | Integration in organizational policies  Clarity Communication & collaboration |
| Chen et al.(38) | 2009 | Remote critical care program network across multiple intensive care units (ICUs) | To discuss and evaluate whether a remote critical care program network can improve clinical and economic performance across multiple ICUs | 63 hospitals in Zhejiang Province | A before-and-after study | Governmental policies and regulations  Bottom-up vs. Top-down |
| Chen (42) | 2018 | Medical device-related pressure ulcer (MDRPU) management modes AS/NZS 4360:2004 risk management standard | To analyse medical device-related pressure ulcer (MDRPU) management modes and their possible risks and provide references to treat MDRPUs | Zhongshan Hospital, Xiamen city, Fujian Province | A before-and-after study | Governmental policies and regulations |
| Cheung et al.(21) | 2011 | Innovative midwife-led normal birth unit (MNBU) | To increase the normal birth rate, thereby decreasing the rate of caesarean sections | Hangzhou First People’s Hospital, Zhejiang Province | A mixed-methods study (retrospective cohort survey, interviews) | Culture fit Doctor-patient relationship  Relative advantage Self-efficacy |
| Cong et al.(109) | 2012 | Quality improvement programs | To determine whether a continuous quality improvement program would reduce pressure ulcer prevalence and to compare PU prevalence before and after implementation of a continuous quality improvement program | A teaching hospital Wuhan, Hubei Province | A before-and-after study | Training & education Feedback |
| Yi Dai (89) | 2018 | Magnet Hospital Model including transformational leadership; structural empowerment; exemplary professional practice; new knowledge, innovations, & improvements; and empirical outcomes) | To present the procedures of developing, implementing, and evaluating of a tailored intervention program towards applying the Magnet Hospital Model in a Chinese hospital and to improve nurses' work engagement | A tertiary hospital, Kunming, Yunnan Province | One-group pretest-posttest of quasi-experiment | Workplace culture  Strategic fit Adaptation Bottom-up vs. Top-down |
| Du et al.(78) | 2014 | Clinical pathways for acute coronary syndromes—phase 2 (CPACS-2) | To provide rigorous evidence to inform the routine use of clinical pathways in the management of acute coronary syndromes in China | 75 hospitals throughout China | A cluster randomized, controlled trial | Human resources Adaptation |
| Du et al.(125) | 2015 | Usual source of care (USC) refers to the provider or place a patient consults when sick or in need of medical advice | To fill this gap in the literature by examining the quality of primary care provided between those having a USC and those without | Guangdong Province | A cross-sectional survey | Health system stakeholders Feedback |
| Fang et al.(126) | 2019 | Home-based cardiac tele-rehabilitation (HBCTR) for coronary heart disease | To investigate whether there was a difference in outcomes following a short-term 6-week HBCTR program for patients | The First Affiliated Hospital of Shantou University Medical College | A parallel two-arm randomized controlled trial | Relative advantage |
| Fu et al.(39) | 2010 | Practical guidelines for community nursing management | To establish standardized management systems for community nursing  To define the roles, responsibilities and qualifications of community nurses  To set up and streamlining various task-oriented nursing procedures for community nurses | The Zhejiang Province | Questionnaire-based surveys and a literature review | Governmental policies and regulations |
| Gao et al.(90) | 2018 | The balanced scorecard | To establish an evaluation indicator system for Guangxi county hospitals and to generate recommendations for hospital development and policymaking. | Five county hospitals, Guangxi | A delphi study | Adaptation |
| Gong et al.(61) | 2018 | Community-based colorectal cancer (CRC) screening program | To describe the implementation of a comprehensive approach to CRC screening in Shanghai, China | Shanghai | A descriptive study | Integration in organizational policies Human resources Motivation  Social demographics |
| Gu et al.(105) | 2011 | Midwives-involved birth care | To explore and describe midwives’ experiences of providing continuity of care to labouring women in the city of Shanghai in China | The labour unit of Fudan University Obstetrics and Gynaecology Hospital, Shanghai | Qualitative open-ended interviews | Leadership |
| Gu et al.(127) | 2015 | Virtual beds pre-hospitalization mode based on business process reengineering and appropriateness evaluation protocol | T o explore optimization of diagnosis and treatment procedure and implementation of virtual beds pre-hospitalization mode for reducing average hospital stay before clinical operation,decreasing medical expenses, and improving the management efficiency of hospital | Ruijin Hospital North， Shanghai Jiao Tong University School of Medicine, Shanghai | A controlled experiment | Relative advantage |
| Hao et al.(110) | 2016 | The CCC-ACS project (a national hospital-based quality improvement program)  ACS: acute coronary syndrome) CCC: cardiovascular disease in China | To improve patient care for ACS through the development and implementation of quality improvement programs | 150 hospital centers across China | A quantitative study | Training & education Feedback |
| By Tsung-Mei Cheng (86) | 2013 | Clinical pathways and case payments | To compare the difference in the cost and quality of services delivered before and after the implementation of clinical pathways To compare the values taken on by a number of variables over time in the pilot hospitals with those in a number of control hospitals not subject to clinical pathways and case payments  To measure the true impact of clinical pathways and case payments on these variables. | Hospitals in 4 Provinces | Ten single controlled trials | Motivation  Perception and attitudes  Feedback |
| Hu et al.(80) | 2016 | The No Pain Labor & Delivery (NPLD) program obstetric anesthesia care | The training NPLD (No Pain Labor & Delivery) To evaluate the effectiveness of our efforts to implement program goals | The Women’s Hospital of Zhejiang University School of Medicine and other 24 additional hospitals. | A before-and-after study | Risk  Motivation  Human resources Training & education Support from relevant actors |
| Huang et al.(128) | 2019 | Family doctors as the main carrier of primary health care | To make a comparison between the contracted and non- contracted residents and make a comprehensive analysis of the Family Doctor and Medical Insurance Payment Coordination Reform | Changning District of Shanghai | A cross-sectional survey | Motivation  Governmental policies and regulations |
| Huang et al.(106) | 2018 | Telepathology (TP) | To share our experience of validating TP for intraoperative frozen section diagnosis in China | Southern Medical University and Guangzhou Huayin Medical Laboratory Center | A retrospective cases review | Simplicity Innovation-workload Costs Governmental policies and regulations |
| Hui et al.(62) | 2014 | The clinical pathway for hospitalized patients with cataract | To investigate the effectiveness of clinical pathway in standardizing medical behaviors, improving work efficiency and quality of hospital management | Xi'an North Hospital, Shanxi Province | A retrospective analysis study | Integration in organizational policies |
| Lei et al. (33) | 2017 | Health information exchange (HIE) projects | To investigate and share the major challenges and experiences of building a regional health information exchange system in China in the context of health reform | Xinjin County, Sichuan province | A desciptive study | Resource scarcity in rural areas Health system stakeholders Human resources Governmental policies and regulations  Adaptation |
| Li et al.(107) | 2018 | PDSA (plan, do, study, action) cycle | To shorten the average length of stay by using ‘Change Acceleration Process’ | Shenzhen People’s Hospital, Guangdong Province | A descriptive study | Leadership |
| Li et al.(34) | 2018 | Family doctor contract services | To determine whether contracting a general practitioner could improve quality of primary care | Three community health centres in Guangzhou, Guangdong Province | A cross-sectional study | Resource scarcity in rural areas Policies and regulations  Strategic fit Training & education |
| Li and Fu (113) | 2014 | The clinical pathway for hospitalized patients with cataract | To evaluate the effectiveness of practicing clinical pathways of patients with cataract in our hospital | Department of Ophthalmology, the Central Hospital of Wuhan, Hubei Province | An observational study | Communication & collaboration |
| Liang et al.(129) | 2019 | General practitioners (GPs) | To discover any vulnerable points of the policy and to provide evidence for advancing a wider range of gatekeeping systems and for perfecting the healthcare system | Five community health centres in Shenzhen and Dongguan, Guangdong Province | A cross-sectional survey | Health insurance  Compatibility Motivation |
| Yi et al.(130) | 2015 | Refined hospital management chain | To analyze the refined management chain of the WuxiNo. 2 People’s Hospital | Wuxi No. 2 People’s Hospital, Jiangsu Province | An observational study | Strategic fit |
| Ren et al.(97) | 2015 | Appropriate healthcare technology (AHT) | To investigate the attitudes of health workers and consumers towards AHTs | Five counties in Zhejiang Province | A quantitative survey | Adaptation Bottom-up vs. Top-down |
| Shen et al.(83) | 2016 | Community-based peer-led diabetic self-management programme | To evaluate a self-management programme collaborating with communities and mobilising peer leaders for patients with diabetes in mainland China | Shanghai | Quasi-experimental nonequivalent control group design | Human resources |
| Shi et al.(131) | 2015 | The ‘gate-keeper’of community health center (CHS) model | To examine which of the dominant primary care delivery models, i.e.,the public CHC model, the ‘gate-keeper’ CHC model, or the hospital-owned CHC models, was most effective in enhancing access to and quality of care for patients with chronic illness | Nine health care organizations in Guangzhou, Dongguan, and Shenzhen cities within Guangdong province | An evaluative study with case-comparison methods | Health insurance  Governmental policies and regulations |
| Wang et al.(100) | 2013 | Quality control circle (QCC) followed the Deming cycle (PDCA cycle) | To use quality control circles (QCC) followed by the PDCA Deming cycle and analyze the application of QCC to the sustained improvement of a medical institution in Zhejiang province | A medical institution in Zhejiang province | An observational study | Bottom-up vs. Top-down |
| Wang et al.(101) | 2018 | Luohu model: A template for integrated urban healthcare systems | To study the integration process, analyze the core mechanisms, and conduct preliminary evaluations of integrated policy development in the Luohu model | Luohu District, Shenzhen | An evaluative study | Communication & collaboration Bottom-up vs. Top-down |
| Wang et al.(91) | 2017 | Quality improvement of lung cancer surgery based on standard operation procedure | To standardize treatment procedure and reduce medical expenses | Beijing Cancer Hospital, Beijing | A retrospective analysis study | Adaptation |
| Wang et al.(87) | 2018 | Multifaceted quality improvement intervention, including a clinical pathway | To determine whether a multifaceted quality improvement intervention can improve hospital personnel adherence to evidence-based performance measures in patients with acute ischemic stroke (AIS) in China | Secondary and tertiary public hospitals with emergency departments and neurological wards | An open-label, cluster-randomized clinical trial | Perception and attitudes |
| Wen et al.(24) | 2017 | In-hospital diagnosis and treatment process in patients with acute ischemic stroke ( AIS) | To analyze the effects of quality supervision and continuous improvement system on optimizing in-hospital diagnosis and treatment process in patients with acute ischemic stroke | Department of Neurosurgery, Changhai Hospital (affiliated to the Second Military Medical University), Shanghai | An observational study | Culture fit Clarity |
| Wu et al.(132) | 2019 | Quality of Care Improvement Initiative (QCI) | To determine whether a clinical pathway–based,multifaceted QCI intervention could improve clinical outcomes among patients with ACS (Acute Coronary Syndrome) in resource-constrained hospitals in China | 101 hospitals across China | A cluster randomized clinical trial | Doctor-patient relationship  Strategic fit |
| Xu et al.(43) | 2018 | PDCA | To minimize adverse respiratory events in our postanesthesia care unit with the guidance of an experienced quality improvement expert from Cincinnati Children's Hospital Medical Center. | Postanesthesia care unit, Shanghai Children's Medical Center, Shanghai | A before-and-after study | Policies and regulations  Clarity Human resources Training & Education Leadership Communication & collaboration |
| Zhang et al.(99) | 2019 | Health XI's results‐based bottom‐up approach | To assess whether Health XI's results‐based bottom‐up approach has effectively incentivized 40 counties to achieve project targets | 40 project counties from eight provinces | A before-and-after study | Bottom-up vs. Top-down |
| Zhang et al.(29) | 2015 | The clinical pathway for pre-hospital CPR (cardiopulmonary resuscitation) | To assess the quality of simulated cardiopulmonary resuscitation (CPR) in local pre-hospital care teams and the improvement achieved by using clinical pathways | Hangzhou Pre-hospital Care Centre, Hangzhou, Zhejiang Province | A prospective observation study | Doctor-patient relationship  Clarity Training & education |
| Zhang et al.(41) | 2019 | Community-based hypertension self-management model with general practitioners | To explore an innovative community-based hypertension self-management model and to evaluate its effects. | Communities of Pudong New Area in Shanghai | A mixed-methods study | Policies and regulations  Support from relevant actors |
| Zheng et al.(69) | 2014 | National Tuberculosis Control Program (NTP) guidelines  Clinical pathway on new smear- positive pulmonary TB inpatients | To evaluate the doctors’ compliance with them in one representative TB hospital for medical quality improvement. | Across China | A retrospective study with medical records analysis | Knowledge and skills Human resources |
| Zhong et al.(92) | 2014 | Dutch-China Cardiovascular Prevention Program (Dutch-China CPP) | To report the evaluation of the implementation of the Dutch-China CPP | Seven communities in Anhui Province | A before-and-after study | Governmental policies and regulations  Adaptation Communication & collaboration |
| Zhou et al.(79) | 2013 | Multifaceted infection control program in reducing ventilator-associated pneumonia | To evaluate the efficacy of an infection control program in reducing VAP in a neonatal intensive care unit (NICU) in China | Neonatal iIntensive care unit, of Children’s Hospital of Fudan University, Shanghai | A before-and-after study | Human resources |
| Ge et al.(95) | 2019 | The clinical pathway of emergency respiratory and cardiac arrest on management of patients with sudden respiratory and cardiac arrest | To explore the effect of clinical pathways of emergency respiratory and cardiac arrest on management of patients with sudden respiratory and cardiac arrest | Huashan North Hospital, Fudan University, Shanghai | A retrospective observational study | Training & education Adaptation Communication & collaboration |
| Lin et al.(44) | 2016 | Guidelines for diabetes in childhood and adolescence | To evaluate the effect of diabetes disease management program (DMP) on glycemic control in type 1 diabetes mellitus (T1DM) patients in Shantou China | Shantou, Guangdong Province | A prospective cohort study | Policies and regulations  Motivation   Awareness Social demographics Training & education |
| Peiris et al.(133) | 2016 | Self-management and interactive technology interventions for T2DM | Protocol for the SMART Diabetes cluster randomised controlled trial; To implement the intervention as  a cluster randomised controlled trial involving 80 communities | 80 communities in one urban (Beijing province) and one rural setting (Hebei province) | A parallel-arm cluster randomized clinical trial | Governmental policies and regulations |
| Feng chun (65) | 2013 | Modification of triage proces for pediatric emergency | To improve the efficiency of pediatric emergency pre-examination and triage, shorten the waiting time of children and improve the satisfaction of children's families | Emergency Department of Shanghai Children's Medical Center Affiliated to Shanghai Jiao tong University, Shang hai | A descriptive study | Integration in organizational policies Human resources Clarity Knowledge and skills Perception and attitudes |
| Guo et al.(81) | 2017 | Modified SBAR communication mode in nursing handover management of patients with severe hepatopathy | To investigate the effect of modified SBAR communication mode in nursing handover management of patients with severe hepatopathy | Department of Hepatopathy, Zhenjiang Third People’s Hospital Affiliated to Jiangsu University, Jiangsu Province | A contolled experiment | Human resources |
| Guo and Sun (134) | 2013 | Quality control circle (QCC) | To explore the methods and effects of quality control circle activities in nursing quality management | North Hospital of Xinjiang Autonomous Region People's Hospital, Nursing Department, Urumqi | A before-and -after study | Simplicity |
| Huang et al.(102) | 2016 | Quality control circle and innovative thinking | To explore the effect of innovative thinking on nursing management | The Affiliated Hopital of Youjiang Medical University For Nationalities, Guangxi | A before-and-after study | Training & education Bottom-up vs. Top-down |
| Ji et al.(103) | 2018 | Nurse scheduling based on six thinking hats thinking model | To explore the application effect of six hinking hats thinking model on improvement of nurse scheduling | Geriatric Endocrinology Department, The First Affiliated Hospital of Nanjing Medical University Nanjing, Jiangsu Province | A before-and-after study | Bottom-up vs. Top-down |
| Liu and Li (70) | 2011 | Foreign five-step method in implementating community nursing | To explore the application of foreign five-step method in community nursing in China | Zaozhuang Traditional Chinese Medicine Hospital, Zaozhuang, Shandong Province | A before-and-after study | Knowledge and skills Leadership Communication & collaboration |
| Long (30) | 2014 | Family doctor system | To understand the implementation of the family doctor system in Shanghai's community, to provide a basis for in-depth construction of community health services | 17 community health service centers, Shanghai | An observational study | Doctor-patient relationship  Human resources |
| Su et al.(98) | 2014 | Delicacy management in the clinical nursing pathway | To effectively apply and implement meticulous management in clinical nursing pathway | Department of Nursing, Wuxi Second Hospital Affiliated to Nanjing Medical University, Jiangsu Province | A before-and-after study | Adaptation |
| Wang (71) | 2018 | PDCA circulation method in the nursing quality management of operating room | To explore the application effect of PDCA circulation method in the nursing quality management of operating room | People's Hospital of Jiaohe City, Jilin Province | A before-and-after study | Knowledge and skills |
| Yang and Wen (93) | 2016 | Modified Early Warning Score (MEWS) | To explore the application effect of the modified early warning score in the comprehensive management of pre-hospital first-aid care | Midong Hospital, Xinjiang Autonomous Region People's Hospital, Urumqi | A prospective group-controlled study | Simplicity Adaptation |
| Zhu et al.(135) | 2013 | Modified Early Warning Score (MEWS) | To evaluate the application effect of the modified early warning scoring system in identifying critical patients in cardiology department | Department of Nursing, Affiliated Hospital of Jiangsu University, Zhenjiang, Jiangsu Province | A non-controlled study | Simplicity Costs |
| Zhu and Qian (72) | 2018 | Modified SBAR communication mode in reversed nursing handover in the oncology clinic | To investigate the efficacy of modified SBAR communivation mode in reversed nursing handover in the oncology clinic | Department of Internal Medicine, Nantong Cancer Hospital, Jiangsu Province | A controlled experiment | Knowledge and skills Training & education |
| Feng et al.(94) | 2019 | Refined management/ Delicacy management | To explore the application effect of the innovative practice of refined nursing management in the management of children's ENT ward | Guangzhou Women and Children's Medical Center, Guangdong Province | A before-and-after study | Adaptation |
| Yang and He (136) | 2014 | Quality control circle | To explore the effect and specific method of practing the quality control circle activities | The Nursing Department of People’s Hospital in Deyang, Sichuan province | A before-and-after study | Motivation |
| Cai et al.(112) | 2020 | Graded nursing based on modified early warning score | To explore the application effect of graded nursing based on modified early warning score in nursing management of orthopedic ward | Guangdong Provincial Workers' Hospital, Guangdong Province | A randomized controlled experiment | Training & education |
| Jin et al.(36) | 2014 | Case management | To explore and establish the holistic working model for nursing based on individual case management | The Nursing Department of Kunshan First People's Hospital, Jiangsu Province | A before-and-after study | Resource scarcity in rural areas Human resources |
| Li (31) | 2015 | Improved family doctor system | To observe and analyze the methods and effects of improved family doctor’s system in the community for managing diabetes patients | The Third Hospital of Yinzhou District, Ningbo, Zhejiang Province | A quantitative study | Doctor-patient relationship |
| Qian (88) | 2016 | Risk management | To explore the clinical value of risk management in cardiology nursing | Yangzhou Hongquan Hospital, Jiangsu Province | A quantitative study | Human resources |
| Zhou (137) | 2017 | Modified nursing record sheet of transfer with the use of SBAR | To explore the application of the modified SBAR nursing record sheet in the nursing risk management of emergency critical patients in hospital transfer | Emergency Department, Wenzhou Central Hospital, Zhejiang Province | A quantitative study | Strategic fit |
| Chen et al.(96) | 2019 | Interdisciplinary care team on the management of Alzheimer’s Disease (AD) | To determine the independent effects of an interdisciplinary care team on the management of patients with AD | The Social Welfare Center in Hangzhou, Zhejiang Province | A single- blind, randomized controlled study | Adaptation |
