# Supplementary material for: Factors Influencing the Implementation of Foreign Innovations in Organization and Management of Health Service Delivery in China: A Systematic Review
Source: Front Health Serv. 2021 Dec 20;1:766677. doi: 10.3389/frhs.2021.766677 (PMC10012679; doi:10.3389/frhs.2021.766677)
Supplement: Supplementary file 3 [file Table_3.docx]

Supplementary material 4: The types of innovations identified

| **Type of Innovations** | **Innovation** | **References** |
| --- | --- | --- |
| Evidence-based best practices | Evidence-based chronic disease prevention (EBCDP) | (48, 82) |
|  | Evidence-based best practices for discharge planning | (77) |
|  | Evidence-based best practices for the management of stoma among colorectal cancer patients | (47) |
|  | Evidence-based best practices in the neonatal intensive care units (NICU) | (54) |
|  | Evidence-based best practices for the management of non-adherence to fluid intake restrictions in hemodialysis (HD) patients | (66) |
|  | Evidence-based practices for nursing management of post-stroke dysphagia | (67) |
|  | Discharge planning for acute coronary syndrome patients | (55) |
|  | International Nosocomial Infection Control Consortium (INICC) multidimensional hand hygiene approach | (51, 52) |
|  | Evidence-based management of cancer related fatigue (CRF) | (50) |
|  | Evidence-based best practice of managing breast cancer patients | (68) |
|  | Evidence-based best practice of management of chemotherapy-induced peripheral neuropathy | (56) |
|  | Evidence-based best practices in clinical handover in a pediatric setting | (57) |
|  | Evidence-based practices for postpartum women in managing perineal care | (58) |
|  | Evidence-based practices of nursing care for transradial angiography and intervention | (59) |
|  | Evidence-based best practices of nursing care for laryngectomy patients | (49) |
|  | Evidence-based pain management practices | (124) |
|  | The Evidence-based Practice for Improving Quality (Quality improvement programs) | (60) |
|  | Community-based colorectal cancer screening program | (109) |
|  | The CCC-ACS project (a national hospital-based quality improvement program) ACS: acute coronary syndrome CCC: cardiovascular disease in China | (110) |
|  | The No Pain Labor & Delivery (NPLD) program | (80) |
|  | Quality improvement of lung cancer surgery based on standard operation procedure | (91) |
|  | In-hospital diagnosis and treatment process in patients with acute ischemic stroke | (24) |
|  | Quality of Care Improvement Initiative (QCI) | (132) |
|  | Health XI's results‐based bottom‐up approach | (99) |
|  | Dutch-China Cardiovascular Prevention Program | (92) |
|  | Multifaceted infection control program in reducing ventilator-associated pneumonia | (79) |
|  | Modification of triage process for pediatric emergency | (65) |
|  | The Joanna Briggs Institute Practical Application of Clinical Evidence System (JBI-PACES) and Getting Research into Practice | (47, 49, 50, 53, 54, 56-59 ,66-68, 77) |
| Integrated care approach | ‘Mutual referral’ between urban community health services and hospitals | (13) |
|  | Clinical pathways | (14, 23, 29, 62, 69, 74, 78, 85-87, 95, 113) |
|  | Integrated traditional birth attendants (TBAs) and skilled birth attendance (SBA) | (32) |
|  | HIV/AIDS case management | (40) |
|  | An integrated care model, namely the Joint Health Center for Chronic Care | (63) |
|  | Integrated health organization reform | (104) |
|  | Innovative care for chronic conditions | (45) |
|  | Innovative midwife-led normal birth unit (MNBU) | (21) |
|  | Magnet Hospital Model | (89) |
|  | Midwives-involved birth care | (105) |
|  | Luohu model: A template for integrated urban healthcare systems | (101) |
|  | Foreign five-step method in implementating community-based nursing | (70) |
|  | Case management | (36) |
|  | Interdisciplinary care team on the management of Alzheimer’s Disease (AD) | (96) |
|  | Dual practice (DP) or multiple job‐holding | (37) |
| Family doctors | Gatekeeping (Family doctor system) | (13, 25, 30, 31, 108, 128, 129, 131 ) |
|  | Family doctor contracting services | (26, 34, 35) |
|  | Usual source of care (USC) refers to the provider or place a patient consult when sick or in need of medical advice | (125) |
| Self-management | Chronic disease self-management program (the Happy Life Club™) | (28) |
|  | Community-based peer-led diabetic self-management program | (83) |
|  | Community-based hypertension self-management model with general practitioners | (41) |
|  | Self-management and interactive technology interventions for T2DM | (133) |
| Health IT | Appropriate healthcare technology (AHT) | (97) |
|  | US electronic health records (EHR) | (84) |
|  | The electronic medication monitor (EMM) | (73) |
|  | Mobile Health Management Service (MHMS) | (76) |
|  | Remote critical care program network across multiple intensive care units (ICUs) | (38) |
|  | Home-based cardiac tele-rehabilitation (HBCTR) for coronary heart disease | (126) |
|  | Telepathology (TP) | (106) |
|  | Health information exchange (HIE) projects | (33) |
| Tools | PDSA plan-do-study-act cycle | (44 ,107) |
|  | The balanced scorecard (BSC) | (75, 90) |
|  | Heart failure discharge checklist | (53) |
|  | PDCA (plan-do-check-act) cycle | (43, 71, 122) |
|  | Home-based and remote sensing rehabilitation protocol among cardiovascular patients | (123) |
|  | Business process reengineering (BPR) | (108) |
|  | Sstandardized postoperative handover protocol (included a postoperative handover checklist) | (64) |
|  | AS/NZS 4360:2004 risk management standard | (42) |
|  | Practical guidelines for community nursing management | (39) |
|  | Guidelines for diabetes in childhood and adolescence | (44) |
|  | Modified SBAR communication mode | (68, 77, 133) |
|  | Modified Early Warning Score (MEWS) | (93, 112, 135) |
|  | Quality control circle (QCC) | (100, 102 134, 136) |
|  | Virtual beds pre-hospitalization mode based on business process reengineering and appropriateness evaluation protocol | (127) |
|  | Risk management | (88) |
| Concept-based approaches | Refined hospital management chain | (130) |
|  | Nurse scheduling based on six thinking hats thinking model | (103) |
|  | Delicacy management in the clinical nursing pathway | (98) |
|  | Refined management (or Delicacy management) | (94) |
