# Supplementary material for: Factors Influencing the Implementation of Foreign Innovations in Organization and Management of Health Service Delivery in China: A Systematic Review
Source: Front Health Serv. 2021 Dec 20;1:766677. doi: 10.3389/frhs.2021.766677 (PMC10012679; doi:10.3389/frhs.2021.766677)
Supplement: Supplementary file 4 [file Data_Sheet_1.docx]

Online supplementary material 1

Searching strategies per database: (date: 02-27-2020)

**embase.com**

('innovation'/de OR (innovati* OR ((state-of-art OR state-of-the-art OR reform* OR improv*) NEAR/6 (behav* OR routine* OR way-of-work* OR ways-of-work* OR Process* OR organization* OR organisation* OR practice* OR idea* OR approach* OR perform* OR hospital* OR public-health*)) OR (reform* NEAR/3 (healthcare OR care OR financial* OR health OR national*)) OR ((new OR novel) NEXT/1 (behav* OR routine* OR way-of-work* OR ways-of-work* OR Process* OR organization* OR organisation* OR practice* OR idea* OR approach* OR perform* OR polic*)) OR (chang* NEAR/6 (routine* OR way-of-work* OR ways-of-work* OR Process* OR organization* OR organisation* OR practice* OR idea* OR approach* OR perform*))):ab,ti) AND ('implementation science'/de OR 'health personnel attitude'/exp OR (implement* OR spread OR adapt* OR adopt* OR uptake* OR scale-up* OR applica* OR facilitator* OR barrier* OR pilot OR stakeholder* OR challeng* OR diffusion* OR enduran* OR dissemination* OR promot* OR reinforce* OR tailor* OR reconstruct* OR sustain* OR equit* OR efficien* OR accessib* OR productiv* OR attitude*):ab,ti) AND ('health care facilities and services'/exp OR 'health care management'/exp OR 'health care personnel'/exp OR 'health care availability'/exp OR 'health care distribution'/exp OR 'health care quality'/exp OR 'health equity'/exp OR (healthcare OR health-care OR health-centre* OR health-institut* OR health-resource* OR Service*-util* OR health-center* OR hospital* OR public-health OR doctor* OR practitioner* OR physician* OR primary-care OR primary-health* OR community-health* OR (medical NEAR/3 (institut* OR center* OR centre* OR facility*))):ab,ti) AND ('China'/de OR 'Guangxi'/de OR 'Inner Mongolia'/de OR 'Ningxia'/de OR 'Tibet'/de OR 'Xinjiang'/de OR 'Chinese'/de OR (China OR (Chinese NEAR/3 (hospital* OR institute* OR nursing OR health-care OR healthcare)) OR Anhui OR Beijing OR Chongqing OR Fujian OR Gansu OR Guangdong OR Guangxi-Zhuang OR Guizhou OR Hainan OR Hebei OR Heilongjiang OR Henan OR Hubei OR Hunan OR Inner-Mongolia OR Jiangsu OR Jiangxi OR Jilin OR Liaoning OR Ningxia-Hui OR Qinghai OR Shaanxi OR Shandong OR Shanghai OR Shanxi OR Sichuan OR Tianjin OR Tibet OR Xinjiang-Uyghur OR Yunnan OR Zhejiang):ab,ti OR (china OR Chinese):jt OR [Chinese]/lim)

**Medline ALL Ovid**

(Organizational Innovation / OR Health Care Reform/ OR (innovati* OR ((state-of-art OR state-of-the-art OR reform* OR improv*) ADJ6 (behav* OR routine* OR way-of-work* OR ways-of-work* OR Process* OR organization* OR organisation* OR practice* OR idea* OR approach* OR perform* OR hospital* OR public-health*)) OR (reform* ADJ3 (healthcare OR care OR financial* OR health OR national*)) OR ((new OR novel) ADJ (behav* OR routine* OR way-of-work* OR ways-of-work* OR Process* OR organization* OR organisation* OR practice* OR idea* OR approach* OR perform* OR polic*)) OR (chang* ADJ6 (routine* OR way-of-work* OR ways-of-work* OR Process* OR organization* OR organisation* OR practice* OR idea* OR approach* OR perform*))).ab,ti.) AND (Implementation Science / OR Attitude of Health Personnel / OR (implement* OR spread OR adapt* OR adopt* OR uptake* OR scale-up* OR applica* OR facilitator* OR barrier* OR pilot OR stakeholder* OR challeng* OR diffusion* OR enduran* OR dissemination* OR promot* OR reinforce* OR tailor* OR reconstruct* OR sustain* OR equit* OR efficien* OR accessib* OR productiv* OR attitude*).ab,ti.) AND (exp Health Care Facilities, Manpower, and Services / OR exp Health Facilities / OR exp Health Care Quality, Access, and Evaluation / OR exp Health Personnel / OR exp Delivery of Health Care / OR exp Health Services Accessibility / OR exp Quality of Health Care / OR exp Health Equity / OR (healthcare OR health-care OR health-centre* OR health-institut* OR health-resource* OR Service*-util* OR health-center* OR hospital* OR public-health OR doctor* OR practitioner* OR physician* OR primary-care OR primary-health* OR community-health* OR (medical ADJ3 (institut* OR center* OR centre* OR facility*))).ab,ti.) AND (China/ OR Beijing/ OR Tibet/ OR (China OR (Chinese ADJ3 (hospital* OR institute* OR nursing)) OR Anhui OR Beijing OR Chongqing OR Fujian OR Gansu OR Guangdong OR Guangxi-Zhuang OR Guizhou OR Hainan OR Hebei OR Heilongjiang OR Henan OR Hubei OR Hunan OR Inner-Mongolia OR Jiangsu OR Jiangxi OR Jilin OR Liaoning OR Ningxia-Hui OR Qinghai OR Shaanxi OR Shandong OR Shanghai OR Shanxi OR Sichuan OR Tianjin OR Tibet OR Xinjiang-Uyghur OR Yunnan OR Zhejiang).ab,ti. OR (china OR Chinese).jw. OR Chinese.la.)

**Web of science Core Collection**

AB=(((innovati* OR ((state-of-art OR state-of-the-art OR reform* OR improv*) NEAR/5 (behav* OR routine* OR way-of-work* OR ways-of-work* OR Process* OR organization* OR organisation* OR practice* OR idea* OR approach* OR perform* OR hospital* OR public-health*)) OR (reform* NEAR/2 (healthcare OR care OR financial* OR health OR national*)) OR ((new OR novel) NEAR/1 (behav* OR routine* OR way-of-work* OR ways-of-work* OR Process* OR organization* OR organisation* OR practice* OR idea* OR approach* OR perform* OR polic*)) OR (chang* NEAR/5 (routine* OR way-of-work* OR ways-of-work* OR Process* OR organization* OR organisation* OR practice* OR idea* OR approach* OR perform*)))) AND ((implement* OR spread OR adapt* OR adopt* OR uptake* OR scale-up* OR applica* OR facilitator* OR barrier* OR pilot OR stakeholder* OR challeng* OR diffusion* OR enduran* OR dissemination* OR promot* OR reinforce* OR tailor* OR reconstruct* OR sustain* OR equit* OR efficien* OR accessib* OR productiv* OR attitude*)) AND ((healthcare OR health-care OR health-centre* OR health-institut* OR health-resource* OR Service*-util* OR health-center* OR hospital* OR public-health OR doctor* OR practitioner* OR physician* OR primary-care OR primary-health* OR community-health* OR (medical NEAR/2 (institut* OR center* OR centre* OR facility*)))) AND ((China OR (Chinese NEAR/2 (hospital* OR institute* OR nursing OR health-care OR healthcare)) OR Anhui OR Beijing OR Chongqing OR Fujian OR Gansu OR Guangdong OR Guangxi-Zhuang OR Guizhou OR Hainan OR Hebei OR Heilongjiang OR Henan OR Hubei OR Hunan OR Inner-Mongolia OR Jiangsu OR Jiangxi OR Jilin OR Liaoning OR Ningxia-Hui OR Qinghai OR Shaanxi OR Shandong OR Shanghai OR Shanxi OR Sichuan OR Tianjin OR Tibet OR Xinjiang-Uyghur OR Yunnan OR Zhejiang)))

**Cochrane CENTRAL register of trials**

((innovati* OR ((state-of-art OR state-of-the-art OR reform* OR improv*) NEAR/6 (behav* OR routine* OR way-of-work* OR ways-of-work* OR Process* OR organization* OR organisation* OR practice* OR idea* OR approach* OR perform* OR hospital* OR public-health*)) OR (reform* NEAR/3 (healthcare OR care OR financial* OR health OR national*)) OR ((new OR novel) NEXT/1 (behav* OR routine* OR way-of-work* OR ways-of-work* OR Process* OR organization* OR organisation* OR practice* OR idea* OR approach* OR perform* OR polic*)) OR (chang* NEAR/6 (routine* OR way-of-work* OR ways-of-work* OR Process* OR organization* OR organisation* OR practice* OR idea* OR approach* OR perform*))):ab,ti) AND ((implement* OR spread OR adapt* OR adopt* OR uptake* OR scale-up* OR applica* OR facilitator* OR barrier* OR pilot OR stakeholder* OR challeng* OR diffusion* OR enduran* OR dissemination* OR promot* OR reinforce* OR tailor* OR reconstruct* OR sustain* OR equit* OR efficien* OR accessib* OR productiv* OR attitude*):ab,ti) AND ((healthcare OR health-care OR health-centre* OR health-institut* OR health-resource* OR Service* NEXT util* OR health-center* OR hospital* OR public-health OR doctor* OR practitioner* OR physician* OR primary-care OR primary-health* OR community-health* OR (medical NEAR/3 (institut* OR center* OR centre* OR facility*))):ab,ti) AND ((China OR (Chinese NEAR/3 (hospital* OR institute* OR nursing OR health-care OR healthcare)) OR Anhui OR Beijing OR Chongqing OR Fujian OR Gansu OR Guangdong OR Guangxi-Zhuang OR Guizhou OR Hainan OR Hebei OR Heilongjiang OR Henan OR Hubei OR Hunan OR Inner-Mongolia OR Jiangsu OR Jiangxi OR Jilin OR Liaoning OR Ningxia-Hui OR Qinghai OR Shaanxi OR Shandong OR Shanghai OR Shanxi OR Sichuan OR Tianjin OR Tibet OR Xinjiang-Uyghur OR Yunnan OR Zhejiang):ab,ti)

**CNKI**

TI=("创新"+"本地化"+"借鉴"+"引进"+"国外"+"传播"+"扩散"+"推广"+"改良")*("医疗服务"+"卫生服务"+"保健服务"+"健康服务"+"护理"+"临床服务"+"诊疗服务"+"住院服务"+"门诊服务"+"家庭医生"+"全科医生"+"整合服务")*("组织"+"管理") OR KY=("创新"+"本地化"+"借鉴"+"引进"+"国外"+"传播"+"扩散"+"推广"+"改良")*("医疗服务"+"卫生服务"+"保健服务"+"健康服务"+"护理"+"临床服务"+"诊疗服务"+"住院服务"+"门诊服务"+"家庭医生"+"全科医生"+"整合服务")*("组织"+"管理")

**VIP**

[((((((((((题名或关键词=创新 OR 题名或关键词=本地化) OR 题名或关键词=借鉴) OR 题名或关键词=引进) OR 题名或关键词=国外) OR 题名或关键词=扩散) OR 题名或关键词=传播) OR 题名或关键词=推广) OR 题名或关键词=改良) AND (((((((((((题名或关键词=卫生服务 OR 题名或关键词=医疗服务) OR 题名或关键词=保健服务) OR 题名或关键词=健康服务) OR 题名或关键词=护理) OR 题名或关键词=临床服务) OR 题名或关键词=住院服务) OR 题名或关键词=诊疗服务) OR 题名或关键词=门诊服务) OR 题名或关键词=整合服务) OR 题名或关键词=家庭医生) OR 题名或关键词=全科医生)) AND (题名或关键词=组织 OR 题名或关键词=管理)) AND (years:[2009 TO 2020])](http://qikan.cqvip.com/Qikan/search/index?LngMySearHistoryIdGuid=4d54bbe3-185b-447c-8b46-dea41bbf489b)

**WANFANG**

题名或关键词 :（("创新" + "本地化" + "借鉴" + "引进"+ "国外" +"改良"+ "扩散" + "传播" + "推广") * ("医疗服务" + "卫生服务" + "保健服务" + "健康服务" + "临床服务" + "门诊服务" + "住院服务" + "诊疗服务" + "护理" + "整合服务" + "家庭医生" + "全科医生") * ("管理" or "组织" )）
